# Quality of life with cediranib in relapsed ovarian cancer: The ICON6 phase 3 randomized clinical trial

**DOI:** 10.1002/cncr.30657

**Published:** 2017-03-24

**Authors:** Dan P. Stark, Adrian Cook, Julia M. Brown, Michael D. Brundage, Andrew C. Embleton, Richard S. Kaplan, Fharat A. Raja, Ann Marie W. Swart, Galina Velikova, Wendi Qian, Jonathan A. Ledermann

**Affiliations:** ^1^ St. James's Institute of Oncology, Leeds Institute of Cancer and Pathology University of Leeds Leeds United Kingdom; ^2^ Medical Research Council Clinical Trials Unit University College London London United Kingdom; ^3^ Leeds Institute of Clinical Trials Research University of Leeds Leeds United Kingdom; ^4^ Queen's University Kingston Ontario Canada

**Keywords:** chemotherapy, health‐related quality of life, ovarian cancer

## Abstract

**BACKGROUND:**

The ICON6 trial showed that cediranib, an oral inhibitor of vascular endothelial growth factor receptors 1, 2, and 3, improved clinical outcomes for patients with platinum‐sensitive relapsed ovarian cancer when it was used with chemotherapy and was continued as maintenance therapy. This study describes health‐related quality of life (QOL) during the first year of treatment.

**METHODS:**

Four hundred fifty‐six women were randomly allocated to receive standard chemotherapy only, chemotherapy with concurrent cediranib, or chemotherapy with cediranib administered concurrently and continued as maintenance. Patients completed QOL questionnaires until disease progression every 3 weeks during chemotherapy and then every 6 weeks to 1 year. Patients alive with disease progression completed a QOL form 1 year after randomization. The primary QOL endpoint was the global score from the Quality of Life Questionnaire Core 30 (of the European Organization for Research and Treatment of Cancer) at 1 year, with the standard chemotherapy group compared with the concurrent‐maintenance cediranib group.

**RESULTS:**

The rate of questionnaire compliance was 90% at the baseline and 76% at 1 year and was similar across the 3 groups. The mean global QOL score at 1 year was 62.6 points for the standard chemotherapy group and 68.7 points for the concurrent‐maintenance group (+4.5; 95% confidence interval, –2.0 to 11.0; *P* = .18). Sensitivity analyses suggested that this finding was robust to the effect of missing data, and the improvement became statistically significant after adjustments for self‐reported diarrhea.

**CONCLUSIONS:**

The 6th study by the International Collaboration in Ovarian Neoplasm (ICON6) showed a significant improvement in progression‐free survival with cediranib as concurrent and maintenance therapy. No QOL detriment with cediranib was found 1 year after treatment was commenced. The maintenance of QOL along with prolonged cancer control suggests that cediranib has a valuable role in the treatment of relapsed ovarian cancer. ***Cancer* 2017;123:2752‐61**. © 2017 The Authors. *Cancer* published by Wiley Periodicals, Inc. on behalf of *American Cancer Society*. This is an open access article under the terms of the Creative Commons Attribution License, which permits use, distribution and reproduction in any medium, provided the original work is properly cited.

## Abstract 2

The ICON6 randomized controlled trial shows a significant progression‐free survival benefit with the use of cediranib, and this article shows no corresponding detriment to the quality of life. Prolonged cancer control, with quality of life maintained, suggests a role for cediranib in the treatment of relapsed ovarian cancer.

## INTRODUCTION

The effects of new cancer therapies on patients' quality of life (QOL) should be assessed alongside their effects on clinical outcomes.^1^, ^2^ The 6th study by the International Collaboration in Ovarian Neoplasm (ICON6) was a phase 3 trial testing the addition of cediranib, an angiogenesis inhibitor, to standard platinum‐based chemotherapy for ovarian cancer. Angiogenesis is an important part of preclinical and clinical cancer growth.^3^, ^4^ Vascular endothelial growth factor (VEGF) is a key mediator of angiogenesis. Inhibition of vascular endothelial growth factor receptors (VEGRs) can prolong survival for patients with ovarian and other cancers.^5^ Cediranib, a tyrosine kinase inhibitor, is an orally bioavailable inhibitor of VEGF signaling through the VEGR1, VEGR2, and VEGR3 receptors, and it has been studied in the management of several tumors.^6^, ^7^, ^8^, ^9^

All patients in ICON6 had relapsed platinum‐sensitive epithelial ovarian, fallopian tube, or primary peritoneal carcinoma (subsequently called ovarian cancer). In the reference group (group A), patients received 18 weeks of standard chemotherapy; in the first intervention group (group B), cediranib was added for the duration of the chemotherapy only; and in the second intervention group (group C), cediranib was given with chemotherapy, and this was followed by maintenance cediranib. Hereafter, the 3 randomized groups are called the reference (group A), concurrent (group B), and maintenance groups (group C). The trial was redesigned by the Trial Management Group after AstraZeneca's decision to cease cediranib development. The redesigned trial required fewer patients; the primary endpoint became progression‐free survival instead of overall survival, with the primary comparison made between groups A and C.

Group C had significantly lengthened progression‐free survival in comparison with group A (hazard ratio, 0.57; 95% confidence interval, 0.44‐0.73; log‐rank *P* < .001).^10^ Improved overall survival was also seen in group C, but the difference was not statistically significant (hazard ratio, 0.76; 95% confidence interval, 0.55‐1.05; *P* = .1), whereas adverse events, including diarrhea and hypertension, occurred more frequently among patients receiving cediranib. A health‐related QOL assessment provides a broad evaluation of a patient's function, well‐being, and symptoms over time. We reported outline QOL data in the initial article on survival; here, balancing cancer control against the nature of the time gained and using validated self‐report measures, we report detailed QOL data describing the wider impact on patients.^11^, ^12^ Previous QOL analyses in large ovarian cancer treatment trials have shed substantial light on this balance.^13^, ^14^, ^15^, ^16^, ^17^, ^18^, ^19^ We report a QOL substudy, designed at the outset of ICON6, that examined key a priori hypotheses about the effect of adding cediranib to standard chemotherapy during the first year of treatment.

## MATERIALS AND METHODS

### ICON6 Parent Study

ICON6 was a randomized, double‐blind, placebo‐controlled, 3‐group (2:3:3 ratio), multicenter, phase 3 Gynecological Cancer Intergroup trial designed to evaluate the safety and efficacy of adding oral cediranib (20 mg daily; AstraZeneca, United Kingdom) to standard platinum‐based chemotherapy. The recommended chemotherapy was carboplatin dosed to target an area under the concentration versus time curve of 6 (AUC6) every 3 weeks in combination with paclitaxel (175 mg/m^3^). Other platinum‐based regimens were allowed if previous toxicity or fitness prevented combination therapy.

After radiological confirmation of relapsed ovarian cancer, 456 women were recruited between November 2008 and December 2011. Treatment in each group was initially planned for 78 weeks (18 months), but a protocol amendment allowed patients to continue treatment indefinitely if they continued to benefit. Each participant gave informed consent, and the study was approved by relevant ethics committees and research governance authorities. The trial is registered with the International Standard Randomised Controlled Trial Number registry (ISRCTN68510403), ClinicalTrials.gov (NCT00532194), and the Australian New Zealand Clinical Trials Registry (ACTRN1261000016003).

### ICON6 QOL Substudy

#### Data collection

Quality of Life Questionnaire Core 30 (QLQ‐C30) and Quality of Life Questionnaire for Ovarian Cancer 28 (QLQ‐OV28) from the European Organization for Research and Treatment of Cancer provided QOL estimates within the preceding 7 days. The QLQ‐C30 contains 30 items, including a global health status scale, 5 function scales (physical, role, emotional, cognitive, and social), and 9 symptom scales/items (fatigue, nausea/vomiting, pain, dyspnea, insomnia, appetite loss, constipation, diarrhea, and financial difficulties). The QLQ‐OV28 contains 28 items focused on ovarian cancer, including abdominal/gastrointestinal symptoms, peripheral neuropathy, chemotherapy side effects (not specifically antiangiogenic‐agent side effects), hormonal/menopausal symptoms, body image, attitude to disease/treatment, and sexual functioning. For each subscale, the score is scaled from 0 to 100. For function scales, high scores indicate better function (improved QOL); for symptom scales, higher scores indicate greater symptoms (poorer QOL). The QLQ‐C30 and the QLQ‐OV28 have undergone extensive psychometric validation and multiple translations and are acceptable to patients.^20^ A between‐groups difference of 10 to 15 points in the QLQ‐C30 global health score was previously defined as having moderate clinical significance.^21^

**Figure 1:**
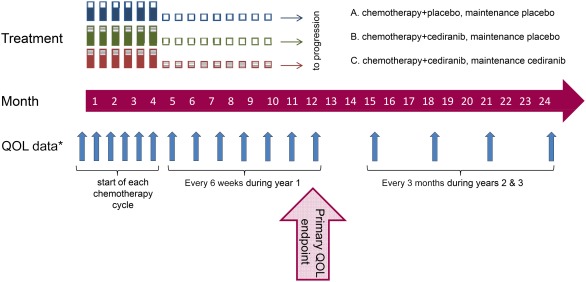
QOL assessed at key time points. *After progression, the QOL assessment was performed at 12 and 24 months only. QOL indicates quality of life.

Questionnaires were completed during outpatient attendance at protocol‐defined time points (Fig. 1). QOL data were collected by patient self‐report, on paper, without conferral with others, and always before medical consultation or treatment administration. QOL data were collected only after disease progression at a scheduled assessment 1 year after enrollment for all patients still alive. Reasons for missing QOL data at scheduled collection time points were requested.

#### Outcomes

The primary QOL endpoint was the QLQ‐C30 global QOL score 1 year after enrollment. The primary comparison was made between groups A and C, which was consistent with survival endpoints. One year was chosen because many patients experience good QOL at that point if they are not receiving chemotherapy and their cancer is controlled. The QOL form closest to 1 year (within ±3 months) was used for the primary outcome.

Secondary outcomes included 3 hypotheses based on cediranib's mechanism for controlling cancer and causing toxicity and on elements of maintenance cancer therapy that can be important to patients.^22^ These were specified a priori to examine the effect of cediranib at different time points during treatment:

Early hypothesis. Angiogenesis is related to ascites formation; therefore, a more rapid resolution of ascites will be observed in patients receiving cediranib. With the abdominal/gastrointestinal subscale of the QLQ‐OV28, the areas under the curve during chemotherapy were compared for group A and groups B and C combined for patients with ascites at enrollment.
Mid hypothesis. The improvement in symptoms will be faster in patients receiving cediranib because of a more rapid reduction in cancer bulk. The changes from enrollment to the midpoint of chemotherapy (the start of cycle 4) in the global, physical‐function, and pain scores of the QLQ‐C30 were compared between group A and groups B and C combined.
Late hypothesis. Maintenance cediranib may continue social impairment and fatigue. The social‐functioning and fatigue scales of the QLQ‐C30 at 1 year were compared between group C and groups A and B combined.

Differences between the treatment groups in all other validated subscales of the QLQ‐C30 and the QLQ‐OV28 were analyzed individually in an exploratory manner with a significance level of *P* = .01.

#### Sample size

The sample size of ICON6 was determined clinically to detect a hazard ratio of 0.65 for progression‐free survival between groups A and C with 2‐sided 5% significance and 80% power. For QOL, this gave us 80% power to detect a small difference between groups (7 points) in global QOL, with the standard deviation estimated from QOL data for the control group of the ICON7 study.

#### Statistical methods

Randomization was performed with permuted blocks stratified by the Gynecological Cancer InterGroup group, first‐line chemotherapy, duration of relapse‐free interval, and previous use of VEGF inhibitors. The statistical significance of the difference in primary QOL outcomes at 1 year between randomized groups was assessed with an analysis of covariance adjusted for the baseline value. Based on repeated measurements from randomization to 1 year, mixed‐effects regression models were used to analyze changes in QOL with unstructured covariance and patient‐level random effects. The statistical significance of secondary outcomes was assessed as follows: for the early hypothesis, an analysis of covariance adjusted for the baseline score; for the mid hypothesis, linear regression modeling of the interaction between the treatment group and whether or not the patient was symptomatic at the baseline; and for the late hypothesis, an analysis of covariance adjusted for the score at the end of chemotherapy. An analysis of covariance was used to compare exploratory outcomes between randomized groups and was also used for a subsequent re‐analysis of the primary outcome with adjustments for diarrhea scores. All analyses were performed with the intention‐to‐treat principle with Stata 13.1.

#### Sensitivity analysis and missing data

To assess the potential impact on the primary outcome of the relatively wide 3‐month visit window, the analysis was repeated with a 1‐month window. To assess the effect of early disease progression, the analysis was repeated after the exclusion of patients whose disease had progressed before 1 year. To assess the effect of patients' stopping treatment early after an adverse event, the primary outcome analysis, adjusted for diarrhea scores, was repeated after the exclusion of these patients.

The potential impact of missing data on the primary outcome was also assessed; imputation was used to model a number of scenarios: In scenario 1, a global score of 0 was imputed for patients who died within 1 year of enrollment. In scenarios 2 to 4, all patients alive at 1 year but missing QOL data were assigned a score; this started with the mean 1‐year score and continued with the mean score minus 10 points and then the mean score minus 20. The rationale for this approach was that patients with missing data may be missing because of illness, and in this case, a lower than average QOL would be expected. In scenarios 5 and 6, control patients alive but missing QOL data were given the mean 1‐year score, whereas maintenance‐group patients were given the mean minus 10 and the then mean minus 20 to examine how much difference would be needed to affect the interpretation of results.

## RESULTS

All ICON6 participants were included in the QOL substudy (n = 456). QOL data were expected from 340 women at 1 year; 72 others had died, 37 were lost to follow‐up after disease progression, and 7 were lost to follow‐up without progression. Baseline data were provided by 410 of the 456 women (90%; Table 1), and 1‐year data were provided by 259 of 340 women (76% of those in follow‐up); 235 of these 340 women (69%) had data for both these time points.

**Table 1:** Completeness of QOL Data by Randomized Group and Time

Phase	Week	Arm A (Reference)		Arm B (Concurrent)		Arm C (Maintenance)		All	
		Patients, No.	QOL, No. (%)	Patients, No.	QOL, No. (%)	Patients, No.	QOL, No. (%)	Patients, No.	QOL, No. (%)
Baseline	0	118	104 (88)	174	158 (91)	164	148 (90)	456	410 (90)
Chemotherapy	9^a^	110	87 (79)	170	102 (60)	163	96 (59)	443	285 (64)
	18^b^	107	71 (66)	165	87 (53)	160	80 (50)	432	238 (55)
Maintenance	39	55	29 (53)	109	49 (45)	118	42 (36)	282	120 (43)
	51^c^	82	63 (77)	126	97 (77)	132	99 (75)	340	259 (76)

Abbreviation: QOL, quality of life.

a Start of cycle 4

b Start of cycle 6.

c QOL data were due from patients who progressed but were alive; there was a 91‐day window for patients who did not progress.

### Primary Outcome

The difference in global QOL 1 year after randomization between patients in groups A and C (the reference and maintenance groups) was neither clinically nor statistically significant (Table 2). The mean 1‐year scores were 68.7 and 62.6 in groups C and A, respectively; the average global QOL was 4.5 points higher in group C than group A (95% confidence interval, –2.0 to 11.0; *P* = .18 after adjustments for the baseline).

**Table 2:** QOL During the First Year of Treatment

	Arm A (Reference)	Arm B (Concurrent)	Arm C (Maintenance)
Follow‐up after 1 y, No.	82	126	132
QOL at baseline and 1 y, No. (%)	55 (67)	89 (71)	91 (69)
Global score, mean (SD)			
Baseline	68.9 (22.4)	73.2 (19.7)	73.3 (18.8)
1 y	62.6 (21.9)	72.5 (21.0)	68.7 (19.7)
Change after 1 y	−6.4 (28.0)	−0.7 (21.7)	−4.6 (20.9)

	Arm B vs Arm A	*P*	Arm C vs Arm A^a^	*P*
Difference in change after 1 y, mean (95% CI)^b^	+8.3 (1.7‐14.9)	.01	+4.5 (−2.0 to 11.0)	.18

	Arm A	Arm B	*P* c	Arm C	*P* d
Change in global QOL, mean (95% CI)^e^					
Baseline to end of chemotherapy	−2.8 (−8.6 to 3.1)	−7.4 (−12.3 to −2.4)	.22	−12.0 (−17.0 to −7.0)	.01
End of chemotherapy to 1 y	−1.0 (−5.4 to 3.5)	5.5 (1.6‐9.3)	.01	2.5 (−1.3 to 6.3)	.14

Abbreviations: CI, confidence interval; QOL, quality of life; SD, standard deviation.

a Primary outcome: 1‐year difference between arms A and C.

b Difference between arms adjusted for the baseline score. Baseline and 1‐year scores for patients with both available were used.

c Arm B vs arm A.

d Arm C vs arm A.

e This repeated measures analysis included patients with data at the baseline and 1 year and used all data for these patients between the baseline and 1 year.

**Figure 2:**
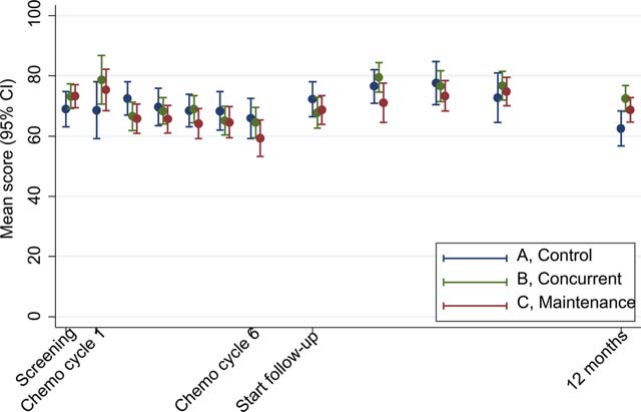
Mean global quality of life in each group, with 95% CIs, over the course of 1 year from study entry. CI indicates confidence interval.

Mixed‐effects modeling of repeated global QOL scores from enrollment to 1 year showed some decline during chemotherapy followed by an improvement during the maintenance period (Table 2 and Fig. 2). The mean QOL changes during chemotherapy were –2.8, –7.4, and –12.0 points in groups A, B, and C, respectively. During maintenance, the mean QOL was reduced further by –1.0 point in group A but increased by 5.5 and 2.5 points in groups B and C, respectively.

### Secondary Outcomes

No significant difference was found between the treatment groups in any of the 3 specific prospectively defined secondary analyses (Table 3). First, among patients with ascites at the baseline, abdominal symptom scores were lower at the end of chemotherapy but by similar amounts for those receiving cediranib and those not receiving cediranib (A vs B/C: *P* = .98). Second, at the midpoint of chemotherapy treatment, global scores, pain scores, and physical‐function scores were all lower than those at the baseline but again by similar amounts for those receiving cediranib and those not receiving cediranib (A vs B/C: *P* for global score = .88, *P* for pain score = .92, and *P* for physical‐function score = .63). Therefore, even in patients with angiogenic processes contributing to symptoms, with platinum‐sensitive relapsed disease, cediranib did not improve patients' reports of symptoms early during treatment. Third, from the end of chemotherapy to 1 year, social‐functioning scores changed little, whereas fatigue scores were reduced similarly among patients receiving and not receiving maintenance cediranib (A/B vs C: *P* for social‐functioning score = .17 and *P* for fatigue score = .71).

**Table 3:** Secondary QOL Outcomes: 3 Cediranib‐Related Hypotheses Defined A Priori

Improved Ascites Resolution During Chemotherapy	Arm A (Reference)	Arms B + C (Concurrent + Maintenance)	P
Patients with ascites present at baseline, No.	39	113	
Abdominal symptom score, mean (SD)			
Baseline	37.1 (25.0)	34.9 (22.0)	
End of chemotherapy	24.2 (19.9)	21.4 (18.2)	.98^a^

Improved QOL (Global, Pain, and Physical Function) by Midpoint of Chemotherapy	Arm A (Reference)	Arms B + C (Concurrent + Maintenance)	*P*
Patients with QOL data at 9 wk and baseline, No.	87	176	
Global score, mean (SD)			
Baseline	69.9 (20.2)	69.1 (21.3)	
Midpoint	64.0 (19.9)	65.8 (19.6)	.88^b^
Pain, mean (SD)			
Baseline	82.7 (19.6)	84.0 (17.9)	
Midpoint	75.0 (20.0)	78.0 (19.0)	.92^b^
Physical function, mean (SD)			
Baseline	23.2 (23.2)	22.0 (24.2)	
Midpoint	20.5 (23.9)	17.8 (22.6)	.63^b^

Impaired Fatigue and Social Functioning During Maintenance Treatment	Arms A + B (Reference + Concurrent)	Arm C (Maintenance)	*P*
Patients with end‐of‐chemotherapy and 1‐y QOL data, No.	140	84	
Social functioning, mean (SD)			
End of chemotherapy	72.0 (27.0)	74.2 (22.0)	
1 y	72.5 (30.8)	78.6 (27.3)	.17^c^
Fatigue, mean (SD)			
End of chemotherapy	37.6 (24.4)	38.0 (22.6)	
1 y	31.0 (25.5)	30.0 (25.2)	.71^c^

Abbreviations: QOL, quality of life; SD, standard deviation.

a Difference in the area under the curve during chemotherapy adjusted for the baseline score.

b Interaction test of the treatment group and whether or not the patient was symptomatic at enrollment.

c Analysis of covariance adjusted for the 18‐week score.

### Exploratory Analysis

The analysis of other subscales of the QLQ‐C30 and the QLQ‐OV28 was exploratory. Most differences between the 3 randomized groups were small and not statistically significant at the prespecified significance level of *P* = .01 (Supporting Tables 2‐4 [see online supporting information]). However, diarrhea was reported more in groups B and C during chemotherapy and continued in group C during the maintenance period. At 12 months, the difference between groups was highly significant (*P* < .001), diarrhea being reported more by patients in group C than those in group A or B. We, therefore, re‐analyzed the primary outcome of global QOL at 12 months post hoc, and we adjusted the analysis for self‐reported diarrhea. The results in groups B and C were very similar, with both having significantly better QOL than group A (Table 4). There was good agreement between self‐reported diarrhea and the clinically reported diarrhea grade (Supporting Fig. 1 [see online supporting information]); it may also be noted that diarrhea was given as a reason for stopping treatment by only 22% of the patients who stopped treatment for toxicity within 12 months.

**Table 4:** QOL During the First Year of Treatment With Adjustments for Self‐Reported Diarrhea

Adjustment for Self‐Reported Diarrhea	Arm B vs Arm A	*P*	Arm C vs Arm A	*P*
Difference in change after 1 y, mean (95% CI)^a^	+7.9 (1.4‐14.4)	.02	+7.4 (0.6‐14.2)	.03

	Arm A	Arm B	*P* b	Arm C	*P* c
Change in global QOL, mean (95% CI)^d^					
Baseline to end of chemotherapy	−3.1 (−8.7 to 2.5)	−3.8 (−8.7 to 1.1)	.84	−7.6 (−12.5 to −2.6)	.22
End of chemotherapy to 12 mo	−3.1 (−7.4 to 1.3)	2.5 (−1.3 to 6.3)	.02	2.8 (−0.9 to 6.5)	.01

Adjustment for Self‐Reported Diarrhea, Excluding Patients Stopping Treatment After Toxicity^e^	Arm B vs Arm A	*P*	Arm C vs Arm A	*P*
Difference in change after 1 y, mean (95% CI)^a^	+6.7 (−0.1 to 13.6)	.05	+10.1 (2.3‐17.8)	.01

	Arm A	Arm B	*P* b	Arm C	*P* c
Change in global QOL, mean (95% CI)^d^					
Baseline to end of chemotherapy	−3.8 (−9.5 to 1.8)	−4.9 (−10.0 to 0.3)	.19	−5.1 (−10.6 to 0.4)	.77
End of chemotherapy to 12 mo	−3.9 (−8.4 to 0.6)	1.6 (−2.5 to 5.8)	.02	3.3 (−0.9 to 7.6)	.01

Abbreviations: CI, confidence interval; QOL, quality of life.

a Difference between arms adjusted for the baseline score. Baseline and 1‐year scores for patients with both available were used.

b Arm B vs arm A.

c Arm C vs arm A.

d This repeated measures analysis included patients with data at the baseline and 1 year and used all data for these patients between the baseline and 1 year.

e Three, 15, and 28 patients in arms A, B, and C, respectively.

### Sensitivity Analysis and Missing Data

Sensitivity analyses did not alter our interpretation of the results. The primary endpoint changed little after the 1‐year visit window was reduced from 3 to 1 months and also after patients whose disease had progressed at 1 year were excluded. Our interpretation was also unchanged after the exclusion of patients who stopped treatment after toxicity, the effect being relatively small after adjustments for the diarrhea score (Table 4).

**Figure 3:**
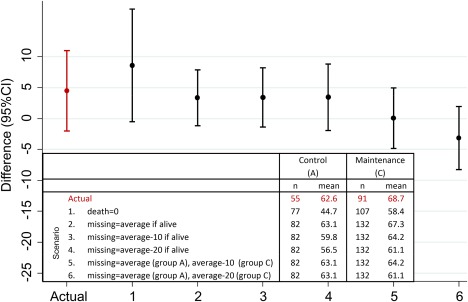
Sensitivity analysis of differences in global quality of life. Point estimates with 95% CIs are shown for the differences between the treatment groups (the Cediranib group [C] mean minus the standard chemotherapy group [A] mean); we imputed a range of postprogression global quality‐of‐life values to estimate the potential impact of missing data. The actual difference and 6 imputed scenarios are shown. CI indicates confidence interval.

The proportion of missing data increased over time (Table 1). At 1 year, QOL data were sought from all surviving patients whether or not their disease had progressed; 81 of 340 forms (24%) were not received (19, 29, and 33 in groups A, B, and C, respectively). The reason for missing forms was known in 31 cases (38%): in 21 of these 31 cases (68%), administrative oversight by the center was the reason; in 6 of these 31 cases (19%), patient refusal not due to illness was the reason; and in 4 of these 31 cases (13%), the patient was too ill. None of the sensitivity analyses modeling the value of missing data through 6 scenarios changed the interpretation of our results (Fig. 3), with the imputation of clinically plausible values used for missing data.^15^ Therefore, our primary finding of no significant decrement in QOL for the cediranib group is robust to the range of clinically plausible unrecorded data with progressive, increasingly treatment‐resistant ovarian cancer.

## DISCUSSION

We report QOL results from the ICON6 trial of cediranib for the treatment of recurrent ovarian cancer. Comparing patients in the reference arm and patients receiving maintenance cediranib treatment, our primary analysis found that global QOL after 1 year was not significantly different according to the intention to treat. We examined whether cediranib improved symptoms early in the treatment course, and this was not the case. Among patients with abdominal symptoms at relapse, the resolution of symptoms during chemotherapy was unchanged with cediranib. Halfway through chemotherapy, there was no difference in pain and physical‐function scores with cediranib. At later time points, no difference was seen in social functioning or fatigue with cediranib

Diarrhea was observed as a significant adverse effect and continued in the maintenance group. With adjustments for self‐reported diarrhea, patients on maintenance treatment had significantly improved QOL in comparison with the reference group. Therefore, although our primary analysis showed global QOL after 1 year to be unaffected by cediranib, the data also suggest that cediranib could provide improved QOL if better diarrhea control could be achieved. This would, however, depend on the quality of diarrhea control at a population level outside clinical trials, such as was achieved, for example, with detailed studies of the physiology of this adverse effect after irinotecan.^23^ Our data indicate the incomplete association between patient reports of diarrhea and clinical severity grading and emphasize the importance of evaluating both aspects of adverse effects.

When the 1‐year period was divided into chemotherapy and postchemotherapy phases, the overall picture was of declining QOL during chemotherapy, with some recovery from the end of chemotherapy to 1 year. There was evidence that in patients receiving cediranib (groups B and C), a greater decline occurred during chemotherapy, but there was also a stronger postchemotherapy recovery. Results in groups B and C were again very similar when they were adjusted for self‐reported diarrhea, and this indicates further the key nature of diarrhea control to the experience of cediranib treatment.

ICON6 is the only randomized trial of cediranib for recurrent ovarian cancer; however, our finding of no QOL detriment tallies with other trials of antiangiogenic therapies (Supporting Table 2 [see online supporting information]), whereas reduced QOL has been reported with erlotinib (an epidermal growth factor receptor inhibitor) as maintenance therapy for recurrent ovarian cancer.^24^ The negative short‐term impact of second‐line chemotherapy plus cediranib contrasts with QOL improvements observed during first‐line chemotherapy combined with antiangiogenic agents.^13^, ^25^ Treating relapsed ovarian cancer before symptom development has previously been found detrimental to QOL,^26^ but 87% of ICON6 patients were symptomatic at randomization.

It is also interesting to note the findings of Stockler et al^15^ in a trial of bevacizumab in recurrent ovarian cancer, where differences were primarily in cancer‐specific symptom scores. This raises the important question whether patient‐reported outcomes studies should focus on cancer‐specific symptom control or whether (in recurrent cancer) the overall impact of treatment on patient function and QOL (balancing toxicity, disruption, and cancer symptoms) is primary when the clinical value of a treatment is being considered. Although this is more difficult to improve (because most treatments have negative aspects), we believe that function and QOL should have primacy in the assessment of palliative cancer therapies as equal partners with estimates of cost, cancer control, survival, and toxicity.^2^, ^27^ Patient‐reported outcome instruments need rigorous development to be valid endpoints in trials, so they will inevitably be available later than clinical symptom assessments for newer drugs with distinct toxicity patterns. Voice changes with cediranib are an example. Therefore, functional and global assessments will retain a key role.^28^

We studied a large group of women representative of the patient population making choices about second‐line therapy for ovarian cancer. The pattern of QOL that we observed, during chemotherapy and afterwards, is consistent with our expectations, and this indicates that the QLQ‐OV28 and QLQ‐C30 instruments are appropriate and sufficiently sensitive for detecting important changes. We achieved a comprehensive collection of QOL data. Any bias arising from missing data should be attenuated by similar levels of missing data across randomized groups. Our sensitivity analyses indicate that our findings are robust to missing data, and this greatly strengthens our interpretation.^29^, ^30^

However, weaknesses include the limited data (5%) from women beyond cancer progression. We chose this design because of patient burden and incomplete contextual data on treatment after relapse. Although sensitivity analyses support our conclusions, missing data were replaced with mean values rather than individual multivariate imputation. We used a wide 3‐month window to estimate 1‐year QOL because of missing data; this adds uncertainty, although a sensitivity analysis applying a 1‐month window did not change our conclusions.

We report no significant difference in global QOL with cediranib among recurrent ovarian cancer patients after 1 year, including a period of maintenance treatment, in comparison with chemotherapy alone. Self‐reported symptom data show that the main toxicity reported by patients with cediranib is diarrhea, and an additional exploratory analysis suggests that improved QOL from the cancer‐control effect of cediranib may be achievable if diarrhea can be better controlled. The primary clinical analysis of ICON6 reported significantly improved progression‐free survival and a nonsignificant improvement in overall survival. The unchanged QOL should be considered alongside the improved clinical outcomes by patients, clinicians, and funding authorities as they consider the case for cediranib as a new therapeutic treatment option for recurrent ovarian cancer.

## FUNDING SUPPORT

The Medical Research Council Clinical Trials Unit at University College London is the lead organization and sponsor of this trial worldwide. This study has also been funded by the UK National Institute for Health Research, Cancer Research UK (CRUK/07/025), the Canadian Cancer Society Research Institute (015469 and 021039), Cancer Australia (APP1006602), the National Gynecological Cancer Centre, and AstraZeneca. The sponsor and all funding sources had a role, through membership in a multiprofessional steering group, in study design, data collection, interpretation, and manuscript preparation along with the authors.

## CONFLICT OF INTEREST DISCLOSURES

Richard Kaplan and Jonathan Ledermann report grants from AstraZeneca and Cancer Research UK. Julia Brown reports a grant from Roche. Galina Velikova reports personal fees from Roche, Novartis, and Eisai outside the submitted work.

## AUTHOR CONTRIBUTIONS

**Dan Stark:** Study design, access to the raw quality‐of‐life data, drafting of the manuscript, interpretation, preparation of the final manuscript, full access to all data, and final responsibility for submitting the manuscript for publication. **Adrian Cook:** Analysis, access to the raw quality‐of‐life data, drafting of the manuscript, interpretation, and preparation of the final manuscript. **Julia Brown:** Interpretation and preparation of the final manuscript. **Michael Brundage:** Interpretation and preparation of the final manuscript. **Andrew Embleton:** Analysis, access to the raw quality‐of‐life data, interpretation, and preparation of the final manuscript. **Richard Kaplan:** Interpretation, access to the raw quality‐of‐life data, and preparation of the final manuscript. **Fharat Raja:** Data collection, interpretation, and preparation of the final manuscript. **Ann Marie Swart:** Study design, interpretation, and preparation of the final manuscript. **Galina Velikova:** Interpretation and preparation of the final manuscript. **Wendi Qian:** Study design, interpretation, and preparation of the final manuscript. **Jonathan Ledermann:** Data collection, interpretation, and preparation of the final manuscript.

## Supporting information

Additional supporting information may be found in the online version of this article.

Supporting Information

Supporting Information
