# Supplementary material for: Quality of life with cediranib in relapsed ovarian cancer: The ICON6 phase 3 randomized clinical trial
Source: Cancer. 2017 Mar 24;123(14):2752–61. doi: 10.1002/cncr.30657 (PMC5516140; doi:10.1002/cncr.30657)
Supplement: Supplementary file 1 — Supporting Information [file CNCR-123-2752-s001.pptx]

## Slide 1
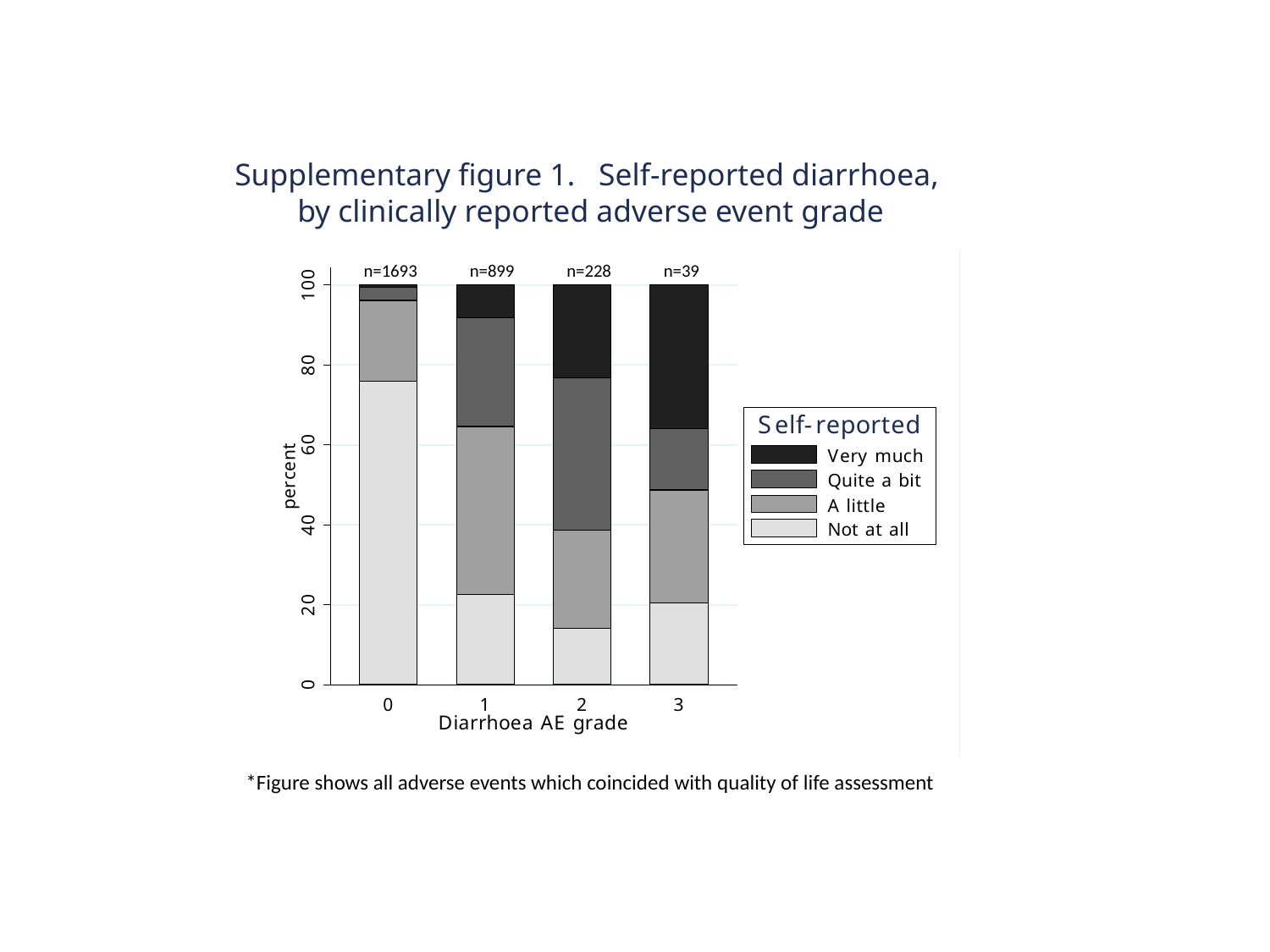

Supplementary figure 1. Self-reported diarrhoea,
by clinically reported adverse event grade
n=1693
n=899
n=228
n=39
*Figure shows all adverse events which coincided with quality of life assessment
