# Supplementary material for: Quality of life with cediranib in relapsed ovarian cancer: The ICON6 phase 3 randomized clinical trial
Source: Cancer. 2017 Mar 24;123(14):2752–61. doi: 10.1002/cncr.30657 (PMC5516140; doi:10.1002/cncr.30657)
Supplement: Supplementary file 2 — Supporting Information [file CNCR-123-2752-s002.docx]

**Supplementary Table 1: Phase III randomised controlled trials of antiangiogenic agents in recurrent Ovarian Cancer**

|  | Design |  | Cohort | Results |  | Patient Reported Outcome Measures (PROMS) |
| --- | --- | --- | --- | --- | --- | --- |
| Trial | Groups | Allocation | platinum-sensitivity of cohort at entry | Primary outcome | Secondary outcome | PROM outcome and finding |
| ICON 6 | Platinum-based chemotherapy + Cediranib or placebo   1. Placebo throughout 2. Cediranib concurrent placebo maintenance 3. Cediranib concurrent plus maintenance | Double-blind, placebo controlled | Platinum-sensitive (100% >6-months prior platinum) | PFS prolonged | OS immature, not prolonged | No detriment to QoL with cediranib as concurrent and maintenance |
| GOG0213 (CP arms) | Carboplatin/paclitaxel +/- bevacizumab followed by bevacizumab and secondary cytoreduction | Randomised open-label | platinum-sensitive recurrent ovarian cancer (100% > 6-months prior platinum) | OS improved (p = 0.056) | PFS improved (p < 0.0001) | QoL with Bevacizumab not different by FACT-O TOI; lower physical functioning during combination treatment and recovery during maintenance bevacizumab. |
| OCEANS | Carboplatin and gemcitabine + bevacizumab or placebo | Double-blind, placebo controlled | Platinum-sensitive (100% >6-months prior platinum) | PFS prolonged | OS immature, not prolonged | Awaited |
| AURELIA | Non-platinum chemotherapy (investigator choice)  +/- bevacizumab | Randomised open-label | Platinum-resistant  (100% <6-months prior platinum) | PFS prolonged | OS not prolonged | Significantly greater improvement in disease-related symptoms and function with bevacizumab[^12^](#_ENREF_12) |
| TRINOVA-1 | Paclitaxel + trebananib or placebo | Double-blind placebo-controlled | Mixed - <12-months prior platinum | PFS prolonged | OS immature, not prolonged | No detriment in global QoL with trebananib^[37](#_ENREF_37)^ |

**Supplementary Table 2. EORTC QLQ-C30 global quality of life and functional scales**

|  | Baseline | | Chemotherapy | | Maintenance | | 12 month^a^ | | p^b^ |
| --- | --- | --- | --- | --- | --- | --- | --- | --- | --- |
| Completed forms | n | | n^c^ | | n^d^ | | n | |  |
| Arm A | 104 | | 112 | | 90 | | 63 | |  |
| Arm B | 158 | | 167 | | 123 | | 97 | |  |
| Arm C | 148 | | 151 | | 110 | | 99 | |  |
|  |  |  |  |  |  |  |  |  |  |
|  | mean | (sd) | mean | (sd) | mean | (sd) | mean | (sd) |  |
| Global health status |  |  |  |  |  |  |  |  |  |
| A | 62.6 | (22.7) | 63.5 | (18.0) | 68.3 | (18.3) | 63.4 | (20.4) |  |
| B | 70.3 | (20.4) | 64.5 | (17.0) | 72.9 | (18.2) | 72.9 | (21.1) | 0.045 |
| C | 68.8 | (20.6) | 63.3 | (16.6) | 67.7 | (19.6) | 68.5 | (19.8) |  |
| Physical function |  |  |  |  |  |  |  |  |  |
| A | 81.5 | (19.9) | 76.5 | (19.5) | 80.4 | (19.3) | 78.4 | (21.0) |  |
| B | 83.5 | (18.7) | 77.7 | (16.8) | 82.9 | (18.0) | 84.0 | (19.2) | 0.116 |
| C | 82.3 | (19.0) | 74.8 | (18.6) | 79.7 | (19.7) | 82.1 | (19.1) |  |
| Role function |  |  |  |  |  |  |  |  |  |
| A | 70.4 | (30.7) | 68.0 | (25.1) | 73.5 | (25.8) | 71.8 | (30.4) |  |
| B | 76.6 | (27.4) | 69.0 | (22.8) | 77.0 | (26.8) | 79.6 | (27.4) | 0.322 |
| C | 77.7 | (27.6) | 66.9 | (25.0) | 74.2 | (27.7) | 77.1 | (25.4) |  |
| Emotional function |  |  |  |  |  |  |  |  |  |
| A | 70.9 | (19.3) | 74.2 | (20.2) | 74.3 | (19.9) | 71.1 | (21.2) |  |
| B | 76.5 | (17.1) | 78.7 | (17.4) | 77.1 | (20.4) | 78.2 | (20.1) | 0.178 |
| C | 75.1 | (20.0) | 76.6 | (19.7) | 78.2 | (20.2) | 77.2 | (21.2) |  |
| Cognitive function |  |  |  |  |  |  |  |  |  |
| A | 82.2 | (22.2) | 76.2 | (21.5) | 77.3 | (22.5) | 80.1 | (22.8) |  |
| B | 84.1 | (18.2) | 80.7 | (18.6) | 81.2 | (21.4) | 83.2 | (22.3) | 0.109 |
| C | 81.6 | (25.3) | 78.6 | (21.5) | 78.2 | (23.8) | 80.8 | (20.0) |  |
| Social function |  |  |  |  |  |  |  |  |  |
| A | 73.1 | (28.5) | 69.0 | (24.6) | 72.9 | (27.4) | 70.4 | (32.0) |  |
| B | 78.5 | (24.2) | 72.4 | (22.0) | 80.4 | (24.7) | 80.2 | (27.6) | 0.182 |
| C | 77.0 | (28.5) | 69.6 | (24.8) | 74.8 | (25.7) | 77.8 | (26.2) |  |

- a - within 3 months

b – 3 group ANOVA, difference in score at 12 months adjusted for baseline

c – number of patients with at least one QoL form during chemo

d – number of patients with at least one QoL form during maintenance

**Supplementary Table 3. EORTC QLQ-C30 symptom scales**

|  | Baseline | | Chemotherapy | | Maintenance | | 12 month | | p |
| --- | --- | --- | --- | --- | --- | --- | --- | --- | --- |
|  | mean | (sd) | mean | (sd) | mean | (sd) | mean | (sd) |  |
| QLQ-C30 |  |  |  |  |  |  |  |  |  |
|  |  |  |  |  |  |  |  |  |  |
| Fatigue |  |  |  |  |  |  |  |  |  |
| A | 33.7 | (24.8) | 37.0 | (21.7) | 29.9 | (21.9) | 31.8 | (24.8) |  |
| B | 28.9 | (22.0) | 37.9 | (19.5) | 27.0 | (21.3) | 27.9 | (24.6) | 0.627 |
| C | 29.3 | (23.3) | 40.3 | (22.0) | 31.4 | (23.9) | 28.3 | (23.5) |  |
| Nausea/Vomiting |  |  |  |  |  |  |  |  |  |
| A | 8.7 | (13.7) | 9.1 | (12.8) | 8.5 | (16.5) | 12.0 | (22.6) |  |
| B | 7.7 | (15.2) | 11.1 | (12.2) | 6.0 | (11.8) | 7.5 | (17.8) | 0.074 |
| C | 9.1 | (16.2) | 12.2 | (14.2) | 7.5 | (12.2) | 5.9 | (14.3) |  |
| Pain |  |  |  |  |  |  |  |  |  |
| A | 25.6 | (27.0) | 19.8 | (20.5) | 22.1 | (23.5) | 20.0 | (25.4) |  |
| B | 24.8 | (25.9) | 19.1 | (18.6) | 18.4 | (23.3) | 19.8 | (26.1) | 0.866 |
| C | 25.1 | (24.5) | 21.0 | (20.5) | 21.4 | (21.6) | 21.2 | (22.1) |  |
| Dyspnoea |  |  |  |  |  |  |  |  |  |
| A | 19.4 | (25.4) | 20.0 | (20.9) | 15.0 | (18.1) | 18.8 | (22.3) |  |
| B | 15.9 | (22.8) | 23.4 | (21.3) | 18.7 | (21.5) | 15.6 | (24.0) | 0.855 |
| C | 16.9 | (23.2) | 26.6 | (22.3) | 18.6 | (22.6) | 19.0 | (27.9) |  |
| Insomnia |  |  |  |  |  |  |  |  |  |
| A | 37.5 | (31.9) | 29.1 | (21.9) | 28.0 | (26.5) | 24.6 | (27.4) |  |
| B | 32.5 | (29.4) | 27.4 | (23.8) | 28.3 | (26.5) | 30.9 | (31.6) | 0.251 |
| C | 30.4 | (27.9) | 27.5 | (22.7) | 23.6 | (24.6) | 26.1 | (30.1) |  |
| Appetite loss |  |  |  |  |  |  |  |  |  |
| A | 18.3 | (27.0) | 13.3 | (20.4) | 13.1 | (24.2) | 14.0 | (21.2) |  |
| B | 19.4 | (27.4) | 20.6 | (21.3) | 10.8 | (17.5) | 14.4 | (27.2) | 0.558 |
| C | 21.8 | (27.5) | 21.6 | (19.3) | 18.5 | (24.3) | 17.2 | (24.5) |  |
| Constipation |  |  |  |  |  |  |  |  |  |
| A | 22.5 | (28.6) | 21.0 | (23.8) | 15.0 | (23.4) | 13.7 | (22.9) |  |
| B | 21.0 | (29.4) | 17.0 | (18.8) | 16.1 | (21.7) | 13.6 | (21.3) | 0.663 |
| C | 18.2 | (26.5) | 16.5 | (18.7) | 9.0 | (14.8) | 10.8 | (21.2) |  |
| Diarrhoea |  |  |  |  |  |  |  |  |  |
| A | 9.2 | (17.0) | 13.3 | (18.8) | 10.3 | (17.6) | 11.8 | (21.8) |  |
| B | 8.6 | (19.2) | 28.1 | (22.6) | 7.4 | (13.6) | 7.9 | (17.9) | <0.001 |
| C | 9.8 | (19.2) | 29.2 | (21.1) | 40.1 | (28.6) | 33.7 | (33.8) |  |
| Financial difficulty |  |  |  |  |  |  |  |  |  |
| A | 17.6 | (29.9) | 15.2 | (24.9) | 14.0 | (25.3) | 15.3 | (29.2) |  |
| B | 15.6 | (26.5) | 14.7 | (22.5) | 14.1 | (25.5) | 10.7 | (23.2) | 0.437 |
| C | 14.3 | (25.0) | 13.1 | (22.0) | 13.7 | (21.9) | 13.1 | (24.2) |  |

**Supplementary Table 4. EORTC QLQ-OV28 symptom scales**

|  | Baseline | | Chemotherapy | | Maintenance | | 12 month | |  |
| --- | --- | --- | --- | --- | --- | --- | --- | --- | --- |
|  | mean | (sd) | mean | (sd) | mean | (sd) | mean | (sd) |  |
| QLQ-OV28 |  |  |  |  |  |  |  |  |  |
|  |  |  |  |  |  |  |  |  |  |
| Abdominal/GI |  |  |  |  |  |  |  |  |  |
| A | 30.1 | (21.4) | 21.9 | (16.3) | 21.4 | (18.9) | 24.8 | (21.4) |  |
| B | 27.1 | (20.9) | 21.6 | (14.4) | 18.4 | (16.3) | 19.2 | (18.6) | 0.065 |
| C | 26.8 | (21.7) | 22.1 | (14.4) | 21.4 | (14.8) | 23.7 | (17.2) |  |
| Peripheral neuropathy |  |  |  |  |  |  |  |  |  |
| A | 11.6 | (20.8) | 30.6 | (25.4) | 24.2 | (25.6) | 22.8 | (24.7) |  |
| B | 9.6 | (17.4) | 24.1 | (21.8) | 24.9 | (25.1) | 20.1 | (25.5) | 0.975 |
| C | 13.1 | (22.7) | 26.8 | (24.4) | 25.6 | (26.7) | 22.7 | (27.2) |  |
| Hormonal |  |  |  |  |  |  |  |  |  |
| A | 18.8 | (24.0) | 21.0 | (23.9) | 20.3 | (25.1) | 18.8 | (24.7) |  |
| B | 22.1 | (28.5) | 18.2 | (23.5) | 19.7 | (25.1) | 18.1 | (26.8) | 0.470 |
| C | 21.4 | (24.1) | 16.7 | (20.3) | 16.5 | (22.0) | 20.2 | (25.6) |  |
| Body image |  |  |  |  |  |  |  |  |  |
| A | 29.2 | (29.6) | 35.8 | (28.3) | 31.5 | (28.5) | 34.4 | (28.1) |  |
| B | 21.3 | (25.5) | 27.9 | (22.7) | 23.9 | (24.5) | 23.3 | (25.2) | 0.016 |
| C | 22.2 | (26.5) | 28.4 | (23.8) | 23.2 | (23.6) | 21.3 | (24.8) |  |
| Attitude to disease |  |  |  |  |  |  |  |  |  |
| A | 47.8 | (27.5) | 47.5 | (23.5) | 40.7 | (24.6) | 46.2 | (26.9) |  |
| B | 42.8 | (26.0) | 41.8 | (21.8) | 35.5 | (25.7) | 30.9 | (24.6) | 0.010 |
| C | 40.6 | (24.7) | 42.8 | (22.4) | 35.9 | (22.6) | 36.2 | (24.8) |  |
| Chemo side effects |  |  |  |  |  |  |  |  |  |
| A | 15.6 | (15.4) | 22.4 | (16.7) | 18.1 | (14.9) | 18.1 | (17.5) |  |
| B | 13.9 | (13.7) | 21.4 | (15.5) | 18.5 | (15.7) | 15.2 | (16.2) | 0.446 |
| C | 15.2 | (15.8) | 22.3 | (16.6) | 18.8 | (19.0) | 18.0 | (20.3) |  |
| Other |  |  |  |  |  |  |  |  |  |
| A | 5.4 | (13.6) | 33.8 | (22.3) | 9.2 | (13.7) | 7.8 | (14.2) |  |
| B | 2.1 | ( 5.9) | 33.1 | (21.7) | 12.5 | (19.5) | 7.0 | (15.8) | 0.964 |
| C | 5.4 | (12.3) | 34.5 | (21.0) | 14.9 | (21.2) | 8.1 | (18.4) |  |

**Supplementary Table 5. Sensitivity analyses.**

A – patients with progression at 12 months excluded

B – 12-month visit window reduced from 3 months to 1 month

|  |  | **Arm A (ref)** | **Arm B (conc)** | **p^a^** | **Arm C (maint)** | **p^a^** |
| --- | --- | --- | --- | --- | --- | --- |
| A | In follow-up at 12 months | n=82 | n=126 |  | n=132 |  |
|  | QoL at baseline and 12 months | 41 (50%) | 67 (53%) |  | 65 (49%) |  |
|  | Global score^b^ |  |  |  |  |  |
|  | baseline | 72.6 (22.1) | 73.3 (19.8) |  | 76.2 (16.3) |  |
|  | 12 months | 62.6 (23.4) | 71.6 (22.0) |  | 70.1 (18.8) |  |
|  | difference in 12-month score |  |  |  |  |  |
|  | vs. Reference^d^ |  | 8.8 (0.9,16.6) | 0.03 | 6.2 (-1.7,14.0) | 0.13 |
| B | In follow-up at 12 months | n=82 | n=126 |  | n=132 |  |
|  | QoL at baseline and 12 months | 49 (60%) | 83 (66%) |  | 89 (67%) |  |
|  | Global score^b^ |  |  |  |  |  |
|  | baseline | 68.0 (21.5) | 72.7 (19.9) |  | 73.2 (18.9) |  |
|  | 12 months | 64.6 (21.9) | 72.5 (21.8) |  | 69.1 (19.6) |  |
|  | difference in 12-month score |  |  |  |  |  |
|  | vs. Reference^c^ |  | 6.0 (-0.9,12.9) | 0.09 | 2.4 (-4.4,9.3) | 0.49 |

a – p-value comparing change with reference group change.

b – mean(sd)

c – difference, mean (95%CI), adjusted for baseline score

-$$--
